# What to Consider When Developing Multidomain Mobile Health Interventions for Lifestyle Management

**DOI:** 10.2196/63573

**Published:** 2025-08-25

**Authors:** Manuel Weber, Renato Mattli, Anja M Raab, Anja Frei, Karin Haas, Thimo Marcin, Albrecht Vorster, Kai-Uwe Schmitt

**Affiliations:** 1 Academic-Practice-Partnership Between School of Health Professions at Bern University of Applied Sciences and University Hospital of Bern, Bern University of Applied Sciences, Murtenstrasse 10, Bern, 3008, Switzerland; 2 Epidemiology, Biostatistics and Prevention Institute, University of Zurich, Zurich, Switzerland; 3 Swiss School of Public Health, Zurich, Switzerland; 4 Institute on Ageing, School of Health Professions, Bern University of Applied Sciences, Bern, Switzerland; 5 Rehabilitation and Sports Medicine, Berner Reha Zentrum, Insel Group, University Hospital of Bern, University of Bern, Bern, Switzerland; 6 Swiss Sleep House Bern, Department of Neurology, University Hospital of Bern, University of Bern, Bern, Switzerland; 7 Interdisciplinary Sleep-Wake-Epilepsy-Center, University Hospital of Bern, University of Bern, Bern, Switzerland

**Keywords:** app, health-related quality of life, lifestyle medicine, mobile phone, nutrition, physical activity, prevention, public health, self-management, sleep, stress management.

## Abstract

Mobile health (mHealth) interventions can transform health care delivery and improve public health. At the same time, the evidence on lifestyle interventions continues to grow. They show promising results in preventing and treating noncommunicable diseases and enhancing health-related quality of life. These factors highlight the potential of multidomain mHealth interventions for lifestyle management. This viewpoint paper focuses on drawing valuable lessons from past experiences and providing guidance to developers of mHealth interventions for lifestyle management. We underscore the critical role of sharing practical insights to advance innovation in the field of mHealth interventions. We used an iterative consensus process to derive lessons learned, identify challenges, and reflect on possible actions. Our insights are based on our experience in developing 2 smartphone-based lifestyle interventions. Challenges and corresponding options in the following areas are presented: target population (preferences, personalization, and delivery), user involvement and testing, human support, and multidomain interventions (interdisciplinarity, flexibility, and core team). The development of multidomain mHealth interventions for lifestyle management requires a participatory and iterative approach involving relevant stakeholders (including end users) so that the right people get the right content at the right time. Additionally, it is crucial to consider established frameworks, guidelines, and regulations; allocate appropriate resources; and form a core team committed to the project’s aims and open to working in an interdisciplinary team.

## Introduction

### mHealth

Globally rising health care costs, the shortage of health care professionals in many countries, and technological progress give mobile health (mHealth) interventions great potential to contribute to public health. In 2024, 4 out of 5 individuals worldwide owned a mobile phone, and about 68% were using the internet, representing an increase of 15 percentage points since 2019 [1]. The World Health Organization defines “mHealth or mobile health as medical and public health practice supported by mobile devices, such as mobile phones, patient monitoring devices, personal digital assistants, and other wireless devices” [2]. mHealth is considered a component or subset of eHealth. mHealth research has evolved from its early inclination to support systems for health services (eg, clinical decision support systems and drug information sources) to more diversified purposes, including preventive care, health promotion, diagnosis, treatment, and monitoring [3].

mHealth interventions have the potential to improve health-related quality of life (HRQoL) at a favorable cost-effectiveness ratio [4,5]. mHealth technologies can enable continuous health monitoring at both individual and population levels, promote healthy behaviors to prevent or mitigate health issues, support self-management of chronic diseases, and provide interventions accessible anytime and anywhere [6]. Despite these opportunities, a recent scoping review [7] identified several barriers associated with the use of mHealth apps. Key issues include problems with validity, usability concerns, technical problems, and data privacy and security concerns. Another systematic review further specified key patient concerns about mHealth apps, including unauthorized access, data breaches, third-party sharing, and lack of transparency [8]. It also identified facilitators, including user education and ensuring data confidentiality and privacy, to enhance trust and adoption. Specifically, for mHealth apps targeting lifestyle behaviors and mental health, maintaining user engagement over time remains a major challenge. Many users stop using these apps after a short period due to technical issues, privacy concerns, poor user experience, and evolving user needs [9]. By improving app design, enhancing usability, personalizing content, and implementing effective user engagement strategies, mHealth interventions may achieve greater long-term adoption and impact.

Evidence on the effectiveness and efficacy of mHealth interventions is growing. In an umbrella review of systematic reviews, positive results of mHealth interventions were reported for chronic disease management, improving chronic pulmonary disease symptoms and heart failure symptoms, reducing deaths and hospitalization, and improving quality of life [10]. In a further umbrella review with meta-analysis, eHealth and mHealth interventions were found to significantly improve physical activity, reduce sedentary behavior, enhance dietary habits, and promote better sleep quality across diverse age and health populations [11]. These findings highlight their potential as scalable and accessible strategies for public health.

### Lifestyle and Health

Lifestyle behaviors emerge globally as strong determinants of morbidity and mortality [9-18]. A recent study showed that adherence to low-risk lifestyle factors was associated with an increased life expectancy at age 30 years [15]. Additionally, different patterns of healthy lifestyles appear to be associated with an increase in the number of years lived without major chronic disease [16]. Furthermore, adopting healthy lifestyles could mitigate the genetic risk of a shorter lifespan [17]. This remains crucial even in later life, as a healthy lifestyle not only reduces the risk of mortality but also contributes to extending life expectancy among older adults [18].

According to the American College of Lifestyle Medicine, lifestyle medicine is a rapidly growing discipline that follows a biopsychosocial approach and rests on 6 pillars: nutrition, physical activity, stress management, restorative sleep, social connection, and avoidance of risky substances [18]. Rooted in these principles, lifestyle interventions center on the individual and take a holistic view of their daily life [19]. Lifestyle interventions have demonstrated substantial potential to enhance HRQoL [20] and extend life expectancy [21]. In recent years, research has increasingly supported these interventions for the prevention and management of noncommunicable diseases (NCDs), such as cardiovascular diseases, respiratory diseases, diabetes, and cancers. Adhering to healthy lifestyle behaviors is an effective [22] and cost-effective strategy for preventing NCDs [23]. The prevention of NCDs could reduce global mortality rates and alleviate sources of morbidity, disability, health care expenditures, and productivity losses [24]. Additionally, lifestyle behaviors are essential factors in the self-management of various NCDs such as cancer [25], cardiovascular diseases [26,27], chronic pain [28], and mental disorders [29].

### Development of mHealth Lifestyle Interventions

Several frameworks, such as IDEAS (Integrate, Design, Assess, and Share) [30] and the updated Medical Research Council framework [31], provide valuable guidance for developing multidomain mHealth interventions. IDEAS represents a step-by-step process to guide the development and evaluation of more effective digital interventions. The updated Medical Research Council framework is designed for developing and evaluating complex interventions. However, these frameworks often operate at a higher, overarching level, with individual steps remaining broad and lacking specificity at a micro level. Hence, it is essential for researchers and health professionals to transparently present their development processes and share insights gained [32]. Current reporting on mHealth interventions and their underlying development processes tends to be insufficient for understanding and replicating those interventions [33].

### Aims

This paper aims to provide guidance to developers of mHealth interventions for lifestyle management. Our focus lies on multidomain lifestyle interventions and their development processes. Specifically, we derive valuable lessons learned, address challenges, and explore opportunities based on our experience in developing 2 multidomain mHealth interventions for lifestyle management. Our focus extends beyond the immediate development process, emphasizing the importance of reflecting on past experiences to inform future digital health initiatives. We recognize the dynamic nature of technology and advocate for adaptability and evolution based on practical experience. Furthermore, we underscore the critical role of sharing practical experiences within the field of mHealth interventions, contributing to its advancement and innovation.

## Methods

To derive lessons learned, identify challenges, and reflect on possible actions, we used an iterative consensus process. Our derived lessons are complementary to the ones of a previous viewpoint paper from 2021 [34] that discusses 5 lessons learned from randomized controlled trials with mHealth interventions. Based on available literature, frameworks, guidelines, and our experience, we identified distinct overarching categories of options. We organized and prioritized them through multiple rounds of discussion, using both inductive and deductive coding strategies, along with mapping techniques. Two authors (MW and RM) categorized the topics, and any discrepancies were resolved through face-to-face discussions and the inclusion of 2 further authors (AMR and KUS) to reach a final consensus. The insights shared in this paper draw from our practical experience in developing 2 smartphone-based interventions, which are detailed in the subsequent sections.

### App-Based mHealth Project Descriptions

Throughout the development processes, we maintained ongoing discussions within the project team about the progress and findings of each step. Our advisory board consisted of experienced health professionals from various fields, including physiotherapy, exercise and sports science, nutrition and dietetics, psychology, nursing, pulmonology, oncology, medical informatics, gerontology, and software development.

#### DELIA

The DELIA (*Digital Lifestyle Intervention for Older Adults*) project aimed to develop and evaluate a digital lifestyle intervention for community-dwelling older adults aged 65 years and older [35]. The project was primarily guided by the IDEAS framework and followed an iterative participatory approach following these key steps:

1. Cross-sectional survey study (phase 1 of IDEAS): At first, a needs assessment for digital lifestyle interventions in Swiss community-dwelling older adults aged 60 years and older was conducted [36]. The goal was to investigate the needs, requirements, and preferences for digital interventions to promote physical activity and additional lifestyle-related content in the target population. A total of 922 respondents with a mean age of 72 years (SD 6.4 years; range 60‐98 years) completed the web-based survey.
2. Semistructured interviews and brainstorming sessions (phases 2‐4 of IDEAS): Interviews with experienced health professionals and researchers from different lifestyle medicine disciplines including sleep, nutrition and dietetics, physiotherapy, exercise and sports science, mindfulness, psychology, gerontology, and software development were carried out to specify target behaviors, identify behavioral strategies, and generate ideas. All experts had experience working with older adults. Their input helped tailor our lifestyle intervention specifically for older adults.
3. Prototype (phase 5 of IDEAS): Based on the previous steps, we developed an initial mock-up of the lifestyle app.
4. Mock-up testing (phase 6 of IDEAS): The mock-up was tested with 6 patients from the patient advisory board of the University Hospital of Bern. They provided valuable feedback on the app’s interface, navigation, and usability.
5. Minimum viable product (phase 7 of IDEAS): Based on the previous steps, we built a fully functional minimum viable product with the most essential features.
6. Pilot testing (phase 8 of IDEAS): Once all app features were implemented, we tested its functionality with project team members during a 1-week testing phase. Subsequently, we performed a pilot test with 6 older adults. Their 2-week testing experience yielded valuable insights that we used to further improve the app. Afterward, we conducted a crossover interventional study (N=108) using a mixed methods sequential explanatory approach to investigate the use and implementation of the mHealth app.

#### QUALUCA

The QUALUCA (*Quality of Life in Lung Cancer Survivors*) project evaluates a digital lifestyle intervention developed for lung cancer survivors. Likewise, this project is primarily guided by the IDEAS framework, whereas the development followed an iterative participatory approach. Key steps included the following:

1. Interdisciplinary workshop (phases 1‐4 of IDEAS): We organized a web-based workshop (4 hours) involving experienced health professionals who work with non–small cell lung cancer (NSCLC) survivors. The goal was to gather insights and experiences from professionals across different disciplines. Their inputs helped tailor our lifestyle intervention specifically for NSCLC survivors by specifying target behaviors, identifying behavioral strategies, and generating ideas.
2. Semistructured interviews and brainstorming sessions (phase 2 of IDEAS): Short interviews with 4 lung cancer survivors who were undergoing inpatient rehabilitation at that time were conducted. These interviews allowed us to better understand their needs and perspectives toward a digital aftercare program.
3. Prototype (phase 5 of IDEAS): Same as DELIA.
4. Mock-up testing (phase 6 of IDEAS): Same as DELIA.
5. Minimum viable product (phase 7 of IDEAS): Same as DELIA.
6. Pilot testing (phase 8 of IDEAS): We performed a pilot test with 3 lung cancer survivors contacted through a national lung cancer survivors’ patient organization. As with DELIA, valuable insights gained from their 2-week testing experience were used to make further improvements to the app.
7. Evaluate efficacy (phase 9 of IDEAS): Currently, we are conducting a randomized controlled trial to primarily assess the efficacy of our digital lifestyle intervention on HRQoL in NSCLC survivors. We are also assessing explanatory outcomes within the intervention group, such as app usability, feasibility, appropriateness, acceptability of the intervention, participant experiences and satisfaction, and app usage data. Detailed information can be found in the published study protocol [37].

## Results and Discussion

In the development of mHealth interventions for lifestyle management, various challenges may arise that complicate the development process and impede progress. Simultaneously, there are also potential courses of action and facilitating factors to address these challenges and ensure smooth processes. The following subsections describe challenges and corresponding options related to the distinct categories identified through the iterative consensus process.

### Challenges and Options Related to Target Population

User preferences and personalization are essential in shaping mHealth interventions. These factors can influence technical implementation and content design. Therefore, user preferences and personalization should be carefully considered in relation to both aspects.

#### Preferences

An understanding of the target population’s characteristics and preferences, including the intervention’s format and layout, is fundamental in the development of mHealth interventions. However, the diverse preferences within target populations present significant challenges [38,39]. Variations in gender, socioeconomic status, cultural background, education, and digital as well as health literacy require prudent and well-considered approaches.

We encourage researchers to conduct initial investigations aimed at understanding the characteristics of their specific target population as proposed in the IDEAS framework. While national statistics and insights from other studies can provide a glimpse into overall trends, a nuanced comprehension of the distinctive attributes of the intended population is essential [40]. To foster engagement, developers must remain mindful of both users and their context. For instance, young people often gravitate toward language and designs that are tailored to their age group [41].

##### Practical Insights

In our projects (DELIA and QUALUCA), both apps allowed participants to adjust the font size, which seems obvious but is crucial for older users. Furthermore, a rather neutral design was chosen with colors high in contrast based on the experience of our interdisciplinary project team with similar target groups. In addition, participants had to specify the frequency of reminders they wanted to receive from the app. The framework MOLD-US [42] was used, which synthesizes barriers influencing mHealth usability for older adults, to inform our decisions in both projects.

We have observed that although the majority of the QUALUCA target population (lung cancer survivors) constitutes a subgroup of DELIA (older adults), they exhibit distinct preferences. For instance, app usage data revealed that DELIA participants often engaged in more activities than the program prescribed. In contrast, preliminary app usage data for QUALUCA participants indicate that the number of prescribed activities tended to be at the upper limit.

Our projects were conducted in Switzerland, where strict data protection regulations apply. Although individual preferences may exist in regard to data protection and privacy, we did not receive any feedback on this topic from participants in our projects. This may be partly because the projects were conducted within the framework of a research study in collaboration with health professionals, potentially fostering a higher level of inherent trust among participants.

### Personalization

Personalization is a “process that changes the functionality, interface, content or distinctiveness of a system to increase its personal relevance” [43]. Research indicates that personalization plays a key role in enhancing user engagement and adherence to mHealth solutions [44]. Additionally, it can positively impact the effectiveness of mHealth interventions [44,45]. Personalization also influences the adoption and utilization of mHealth technology. Rather than adopting a generic “one-size-fits-all” approach, personalized strategies tailor health care services to address the unique needs of each subgroup and individual. In the context of mHealth, personalization can manifest in various ways, including customized messaging, personalized treatment plans, and individualized data analysis [46]. The main purposes of personalized mHealth solutions are disease self-management and promotion of lifestyle behaviors [43].

A metaethnographic review [41] investigating patients’ perceptions of mHealth apps revealed a prominent emphasis on the necessity for greater personalization of app content, as highlighted by numerous participants across these studies. Patients felt that since they are the ones using these apps, they should be able to make them suit their needs better. Therefore, it is important that these apps have different features that let users customize them to fit what they need.

Tailored mHealth interventions appear to be more effective in physical activity promotion among adults than nontailored interventions [47]. However, future research is needed to address the following areas to fully realize the potential benefits of personalization in mHealth: developing design frameworks to increase users’ motivation and engagement, creating guidelines for integrating personalization into mHealth solutions, establishing reporting standards, studying the effects of personalization techniques, and exploring how to adapt accessibility for different user groups [43].

#### Practical Insights

Every individual has their own lifestyle and daily routines with different factors having different levels of importance. In contrast, there are general lifestyle recommendations for different target groups. Based on our experience, personalization is a key aspect to ensure that every individual gets the right content at the right time. For instance, considering the wide variation in initial fitness levels, offering various difficulty levels is essential. Our apps assigned participants to different levels or tracks in the background based on the initial data participants provided upon installation. After the first week and again after the sixth week, participants were automatically asked by the app whether the training intensity was sufficient. Depending on their response, the level was maintained or adjusted by algorithms in the background. Such options for personalization can give users a sense of self-determination.

### Delivery

When considering content delivery, various decisions must be made. For example, determining the format of the content: Should it be delivered through videos featuring subtitles and instructors, audio files with voice-overs in different languages and dialects, or simply text? Preferences vary among individuals; while some may prefer visual aids, others may find audio instructions more convenient. It is also important to choose a linguistic style that is appropriate for each subgroup of the target population [48]. Many users may not be familiar with technical terms, so using too much jargon can confuse them and make the intervention unnecessarily complex.

Besides, integrating gamification elements, such as interactive features, may be a promising approach to enhancing user engagement and motivation [49]. Research indicates that gamification may be particularly effective in healthy and younger adults [50]. However, the level of gamification should be aligned with the preferences and cultural norms of the target population.

#### Practical Insights

Although confronted with financial and time constraints, there was some content delivered in different forms (text and audio) to give the participants the option to choose according to their individual preferences. However, only limited interactive elements could be incorporated into our projects. Overall, the decision was intentionally made to keep the app simple and minimalistic, considering our target populations. Introducing too many elements could overwhelm individuals, especially those with lower digital literacy. Furthermore, several experts from various fields related to the target population reviewed the content repeatedly and provided valuable inputs. This approach facilitated the accumulation of diverse perspectives and the harnessing of expertise from each discipline involved.

### Challenges and Options Related to User Involvement and Testing

Participatory approaches, such as co-design or cocreation, foster user-centered design in mHealth interventions. These methods involve an iterative process in which targeted end users and other relevant stakeholders collaborate with researchers [51]. Ideally, shared decision-making should be maintained throughout the project life cycle. In this context, co-designed mHealth interventions may be more effective than traditional approaches, where interventions are primarily designed by researchers and clinicians [52].

Initiating testing as early as possible is crucial. Using methods, such as paper mock-ups, allows for preliminary assessment even before the product reaches its final stages. This early testing phase aids in identifying potential issues and gathering initial feedback, which can inform subsequent iterations. However, remaining receptive to user feedback throughout the entire development process is vital. Being open to feedback at any stage enables developers to address emerging concerns promptly and make necessary adjustments, thereby enhancing the overall user experience.

Additionally, careful consideration should be given to the selection of participants for testing. By including diverse participants with varying backgrounds, preferences, and levels of expertise, developers can gain comprehensive insights and ensure that the product meets the needs of its intended users.

#### Practical Insights

We began involving various stakeholders early on, such as health professionals and software developers, along with potential end users. This approach helped us better characterize and understand the perspective of the target population. Additionally, throughout the development process, we remained open to feedback at every stage. Even when certain adjustments were no longer feasible due to limited resources, they still contributed valuable insights for future projects. From a more technical point of view, we recommend thoroughly balancing between having an early prototype and specifying the technical implementation.

### Challenges and Options Related to Human Support

In mHealth interventions, human support (digital person-to-person support or face-to-face) can play a role in overcoming various challenges. One key challenge is ensuring that users have access to adequate support when navigating complex health technologies. This includes addressing technical issues, clarifying instructions, and providing guidance on how to effectively use the intervention. Additionally, maintaining user engagement and motivation over time can be challenging, requiring personalized interactions and encouragement from human support personnel. Furthermore, cultural and linguistic diversity among users may necessitate tailored support approaches to ensure inclusivity and effectiveness. Despite these challenges, leveraging human support in mHealth interventions offers opportunities to enhance user satisfaction, improve intervention outcomes, and foster a sense of trust and connection between users and the intervention program [53].

The question of how much human support is truly necessary poses a significant consideration. While providing ample personal support is essential for ensuring users’ adherence [44,54], excessive support can potentially hinder user autonomy and scalability of the intervention. Striking the right balance is crucial, tailoring support levels to the specific needs and capabilities of the target users. Understanding users’ proficiency with technology, health literacy, and individual preferences is key in determining the appropriate level of support. Moreover, using scalable support models, such as tiered support systems or automated assistance (eg, chatbots), can help optimize resource allocation while still meeting users’ support needs effectively. Ultimately, achieving the optimal balance between human support and user autonomy is pivotal in maximizing the impact and sustainability of mHealth interventions.

#### Practical Insights

In the DELIA study, there was no individualized support provided at the beginning of the intervention. Instead, an introductory email was sent to the participants including all the required information to install and use the mHealth intervention. Furthermore, a project-specific email address was provided, and participants were also able to contact the study team directly through the mHealth app. Although some participants required some support for installing the app, the chosen path for implementation was considered appropriate for the target population of this project.

In the QUALUCA study, we conduct a comprehensive onboarding session (90 minutes) at the onset of the intervention. During this web-based onboarding, the app is installed together with the participants. Additionally, the app’s features are explained, and safety-related aspects are discussed. The onboarding aims to ensure that participants use the app effectively and derive maximum benefit from it. Furthermore, it is designed to minimize the need for support during the 12 weeks of intervention. Participants who have already completed the study highly appreciated the onboarding appointment, as it allowed them to address ambiguities and questions directly. Ambiguities and questions could be addressed directly. Additionally, participants have a dedicated contact person available to assist them with any challenges they may encounter. This can build trust in the intervention and the study, as well as enhance overall motivation and adherence. Furthermore, addressing questions raised by participants during onboarding helps gain a better understanding of the target group and how they use the app.

### Challenges and Options Related to Multidomain Interventions

#### Interdisciplinarity

Self-management and lifestyle interventions often use holistic and interdisciplinary approaches. Not only are the topics covered in such interventions interdisciplinary, there are also different theoretical frameworks, pedagogical concepts, and technical solutions that need to be considered [55]. This challenging task asks for a team effort combining different disciplines. To successfully connect people from different disciplines, there needs to be a common understanding of the underlying principles and aim of the intervention. Therefore, we recommend allocating some specific time to build such a common understanding and fundament for future collaboration at the beginning of each project. Although such effort costs time and money, it can pay off during the remainder of the project. Furthermore, we recommend involving various health professionals and other relevant stakeholders through focus group meetings or similar methods to gain insights from different perspectives and to understand the broad context [56].

##### Practical Insights

Highly experienced health professionals and scientists from various backgrounds were involved in the iterative design of our interventions, contributing to both the development of themes and the functionalities of the app. Disciplines covered included sleep, nutrition and dietetics, physiotherapy, exercise and sports science, mindfulness, psychology, and medical informatics. The concept of lifestyle medicine and lifestyle interventions was thoroughly introduced to the entire project team at the beginning of the intervention. We also highlighted the primary aim of the interventions, that is, increasing participants’ HRQoL. We strived to embrace diverse perspectives and foster a shared understanding, particularly crucial in bridging the communication gap between software developers and health professionals.

### Flexibility

The development of multidomain lifestyle interventions demands a high degree of flexibility from the involved professionals. Frequently, professionals must step out of their comfort zones to develop solutions that meet the needs of the target populations while contributing to the overarching project goal. When combining several lifestyle domains in parallel, the time requirement of an intervention increases. This must be offset by the needs and capabilities of the participants and requires professionals to take a step away from their traditional single-domain intervention. However, this calls for project members who see the value in the other lifestyle domains and are willing to change perspectives.

#### Practical Insights

The degree of flexibility of the professionals and project partners involved was carefully considered when setting up the project organization. For example, the overarching project goal was communicated at the outset and the commitment of each professional was a mandatory requirement for collaboration. This can be further illustrated using the example of mindfulness exercises. According to various traditional views, mindfulness exercises should last at least 45 minutes. However, recent research has shown the effectiveness of shorter exercises [57]. Such shorter mindfulness exercises were key for our interventions as the participants were exposed in parallel to content and exercises from other lifestyle domains. Therefore, it was important for us to involve project partners who were open to shorter mindfulness exercises.

### Core Team

The development of mHealth interventions has 2 core aspects: content and technical implementation. Normally, professionals are experienced in one or the other aspect. This requires each core team member, independent from the background, to relinquish control at least partially. This is also related to another key necessity: mutual trust between core team members. We recommend planning enough time for the technical specification and the development of the content of the mHealth intervention. Additionally, it is important to allocate enough time to build trust in interdisciplinary collaboration, especially in new collaborations where team members often do not know each other. Staffing such projects with open-minded personnel is crucial for success. A high degree of openness is required, for example, when assumptions turn out to be incorrect. Furthermore, high adaptability is needed to be able to immediately respond to unexpected feedback or bugs that occur during the development process.

#### Practical Insights

Although we prespecified the technical implementation in detail, we encountered unexpected new features and unforeseen bugs that required rapid adaptation. The challenges of executing research projects under tight time and financial constraints often leave certain aspects open-ended, requiring continuous problem-solving and iteration. To navigate these uncertainties effectively, we prioritized assembling a core team of open-minded, adaptable, and multidisciplinary professionals. Their diverse expertise allowed us to approach challenges from multiple perspectives, enabling faster troubleshooting and innovative solutions.

### Summary

**Figure 1.**
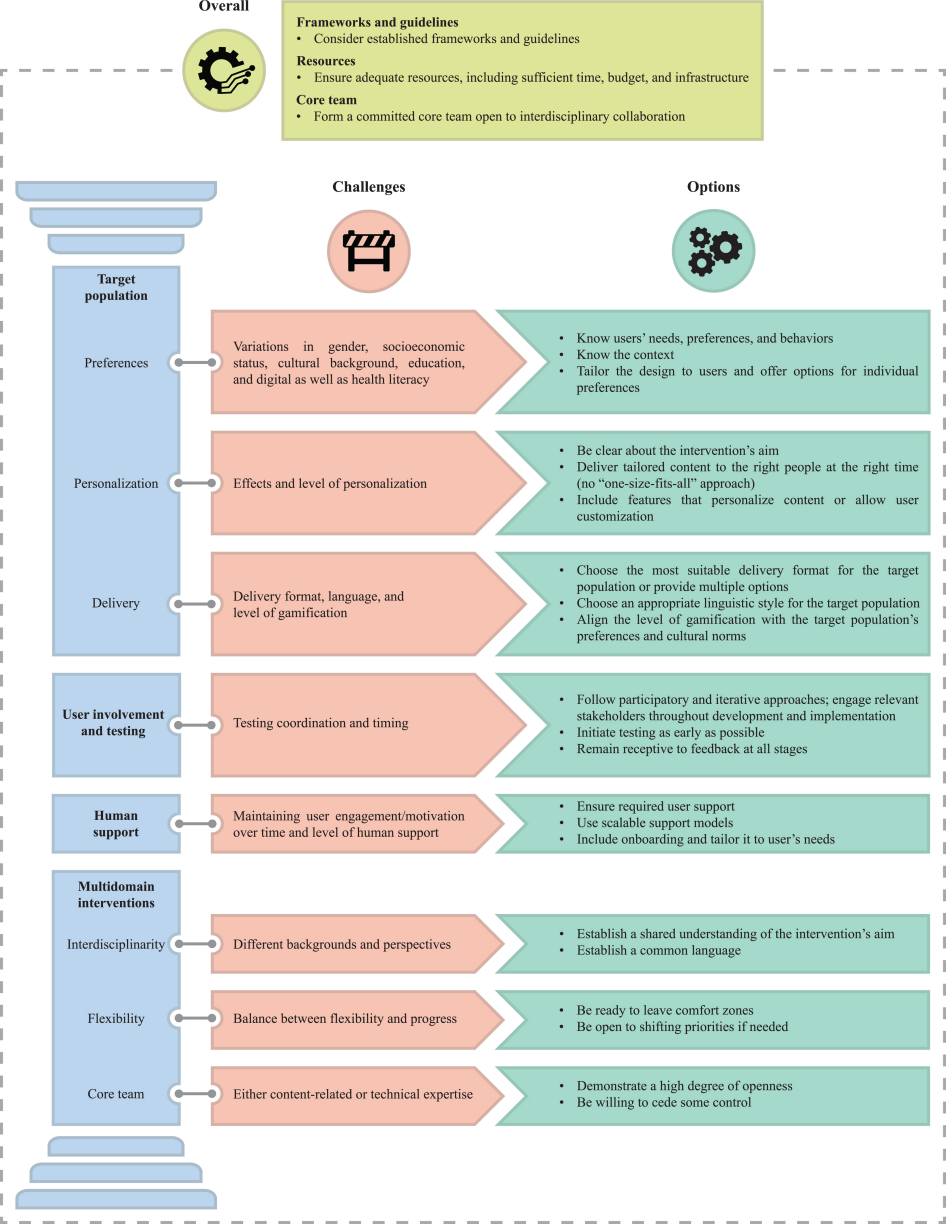
Key considerations in the development of multidomain mHealth interventions.

Figure 1 shows a summary of the key considerations outlined in this viewpoint paper.

### Conclusions

In this viewpoint paper, we discuss the valuable insights gained from the development of multidomain mHealth interventions for lifestyle management. Our investigation encompasses various aspects, including considerations related to the target population, user involvement and testing, human support, and multidomain interventions. Understanding the diverse needs and preferences of the target population is crucial. Factors such as gender, socioeconomic status, knowledge and education, and digital literacy, as well as health literacy, considerably impact user engagement and adherence. Successful multidomain lifestyle interventions deliver the right content at the right time to the right people and, therefore, include features that tailor the content toward the individual’s needs or let users customize them to fit what they need. Furthermore, these interventions require collaboration across multiple disciplines and the involvement of key stakeholders, including end users. Project leaders should establish a common understanding of the intervention’s aim and a common language between the project members early on. Stepping out of comfort zones allows for innovative solutions. Valuing other disciplines rather than centering solely on one's own expertise is essential. Partially relinquishing control, maintaining flexibility, and embracing openness to revising assumptions lead to better outcomes. In summary, the development of multidomain mHealth interventions for lifestyle management demands a holistic and interdisciplinary approach to address challenges and leverage options in the development process.

## Abbreviations

DELIA: Digital Lifestyle Intervention for older Adults
HRQoL: health-related quality of life
IDEAS: Integrate, Design, Assess, and Share
mHealth: mobile health
NCD: noncommunicable disease
NSCLC: non–small cell lung cancer
QUALUCA: Quality of Life in Lung Cancer Survivors
